# Coronary Artery Calcification in Rheumatoid Arthritis Patients: A Systematic Review

**DOI:** 10.7759/cureus.70517

**Published:** 2024-09-30

**Authors:** Stephanie Nagy, Jordan Ditchek, Marc M Kesselman

**Affiliations:** 1 Rheumatology, Kiran C. Patel College of Osteopathic Medicine, Nova Southeastern University, Davie, USA; 2 Radiology, Dr. Kiran C. Patel College of Allopathic Medicine, Nova Southeastern University, Davie, USA

**Keywords:** autoimmune diseases, cardiovascular disease, coronary artery calcification, ct coronary calcium score, rheumatoid arthritis

## Abstract

Rheumatoid arthritis (RA) is one of the leading autoimmune causes of inflammatory arthropathy worldwide. The musculoskeletal impacts of RA are well described within the literature. More recently, research efforts have highlighted that inflammation associated with the condition is not solely isolated to the joint synovium. Specifically, data has demonstrated that the cardiovascular system is negatively impacted by inflammation tied to RA, with adverse cardiovascular outcomes considered the leading cause of mortality among patients with RA. One approach to determine the risk for cardiovascular disease (CVD) is computed tomography (CT) coronary angiography, a noninvasive imaging approach that analyzes the calcifications within the coronary vessels. This has increasingly been utilized to analyze plaque burden and vessel obstruction, which is measured using the coronary artery calcium (CAC) score. A total of 305 articles were analyzed, and 11 articles were selected for this review based on inclusion and exclusion criteria. The results indicated that nearly 60% of patients with RA experienced an elevated CAC score. As such, patients with RA likely carry a higher risk for adverse cardiovascular outcomes as compared to their healthy counterparts. Additional research is warranted based on these findings to determine whether the addition of CT coronary angiography and analysis of laboratory markers for CVD, including lipid markers in standard protocols for RA comorbid assessment, would help to reduce adverse cardiovascular complications.

## Introduction and background

Rheumatoid arthritis (RA) is one of the leading autoimmune causes of inflammatory arthropathy worldwide. It is a chronic and progressive condition that damages the synovial joints, resulting in functional loss due to cartilage and bony erosion as well as further advances into extra-articular disease manifestations [1]. It is estimated that over 17 million individuals have RA, with an estimated global prevalence of 208 cases/100,000 cases [2]. 

The underlying pathophysiology tied to the development and progression of RA is complex and has yet to be fully elucidated. Research efforts to date have shown that underlying mechanisms of disease activity are likely attributable to a combination of genetic alternations, environmental factors, and dysfunctional immunological processes. One mechanism of disease onset and progression involves immune dysregulation tied to T cells. This includes the upregulation of T helper cell type 1 (Th1) and Th17 and the downregulation of T regulatory and Th2 [3-5]. The overexpression of Th1 and Th17 cells induces the expression of interferon-gamma, tumor necrosis factor-alpha (TNF-α), and interleukin (IL)-2 and TNF-α, IL-1, IL-6, and IL-8, respectively, which are all categorized as proinflammatory cytokines that result in joint erosion and cartilage breakdown [3-5]. On the other hand, downregulation of T regulatory cells, which control the level of expression of T-cells within the immune system, results in overexpression of inflammatory Th1 due to the loss of control. Th2 also secretes anti-inflammatory markers of IL-4, IL-5, and IL-13. IL-4 can counteract and reduce the expression of the inflammatory markers expressed by Th1. Meanwhile, IL-4 is diminished in patients with RA [3-5]. The mismatch between proinflammatory and anti-inflammatory immune factors has been tied to the development of RA. In addition, the underlying mechanisms of RA have been shown to be associated with a loss of self-tolerance, which results in B-cells producing antibodies against self-antigens. The two autoantibodies included in the diagnosis of RA include rheumatoid factor (RF) and anti-citrullinated peptide antibodies (ACPA) [4,6]. Patients who test seropositive for RF and/or ACPA tend to have more severe and aggressive disease states and increased inflammation [7-8]

Common symptoms tied to RA presentation are associated with inflammation, especially within the smaller joints of the hands and fingers before progressing to larger joints. Commonly, the synovial joints are impacted by the autoantibody-cytokine-interleukin inflammatory cascade, resulting in inflammation, erythema, warmth, and limited range of motion, which can lead to the development of swan neck or boutonniere deformation of fingers, ulnar deviation, and metacarpal subluxation if the inflammation is not suppressed early [9].

The musculoskeletal symptoms of RA are well-known, but the extra-articular symptoms are not fully understood and can often lead to more serious complications, especially with the involvement of the cardiovascular system. RA is an independent risk factor for cardiovascular disease (CVD). This risk has been exemplified in the rheumatology community by the European League Against Rheumatism (EULAR) task force that brought awareness to the risk of CVD in RA in 2010 and 2015, updating their recommendations most recently in 2022 to include all other rheumatological conditions [10,11]. Most recently, the recommendations surround modifying current cardiovascular prediction tools of the Framingham Risk Score, QRISK3, or Systematic Coronary Risk Evaluation to better fit the risks of these patients as they either have been noted to underestimate or have not be tested completely for their use in this population, in addition to completing regular CVD assessments with lipid panels, the inclusion of plaque screening, providing lipid-lowering medications and lifestyle modifications [10,11]. Recent studies have shown that CVD is the leading cause of mortality among patients with RA and that they have a greater incidence of ischemic heart disease and heart failure as compared with the general population [12,13]. RA patients with active disease states have significantly worsening atherosclerosis disease states, with a 2.5-fold increase in coronary calcification, and have more rapidly progressing atherosclerosis as compared to their patients without RA [14,15].

Invasive coronary angiography is considered the gold standard for diagnosing cardiac abnormalities, but noninvasive computed tomography (CT) coronary angiography is a noninvasive imaging approach that analyzes the calcifications within the coronary vessels and has gained popularity in recent years. This has increasingly been utilized to analyze plaque burden and vessel obstruction due to its low-risk nature and ability to identify calcifications within the plaque, which is measured using the coronary artery calcium (CAC) score. The CAC score is calculated by using a CT scan measuring the calcification buildup within the atherosclerosis plaque in the cardiac vasculature. Calcification is measured via Agatston units to determine the extent of plaque buildup: 0 Agatston units indicate no disease state, 1-99 Agatston units for mild disease, 100-400 Agatston units for moderate disease, and above 400 Agatston units for severe disease [16]. CT coronary angiography has a sensitivity of 94%, specificity of 97%, positive predictive value of 87%, and negative predictive value of 99% [17]. With 43.5% of patients with RA experiencing dyslipidemia due to progressive inflammation, it may be beneficial for patients with RA to undergo CAC scoring to detect atherosclerotic changes, leading to earlier treatments, including lipid-lowering medications and stenting to prevent further progression and complications [18].

In an effort to better understand the risk of CVD tied to RA, we performed a systematic review of the literature using Ovid, CINAHL, Web of Science, and Embase, analyzing the use of CAC in patients with RA. This will help to assess the need to include CAC scoring in the standardized management of patients with RA for early identification and prevention of coronary artery obstruction and complications.

## Review

Methods

Search Strategy

A systematic literature review was performed using CINAHL, Ovid, Embase, and Web of Science using the search terms “Rheumatoid arthritis” AND “Coronary artery calcium score” OR “Coronary Calcification Score” OR “Coronary artery calcification.” To ensure the recency of the articles, only articles published between 2010 and 2024 and available in the English language were assessed. The articles were analyzed in a step-wise process: (1) We evaluated the title and abstract for relevancy, and (2) we assessed the full-text manuscript to ensure that pertinent information on CAC scoring was reported among patients with RA. The Nova Southeastern University (NSU) library database was utilized to access databases and full-text articles.

Selection Criteria

**Figure 1:**
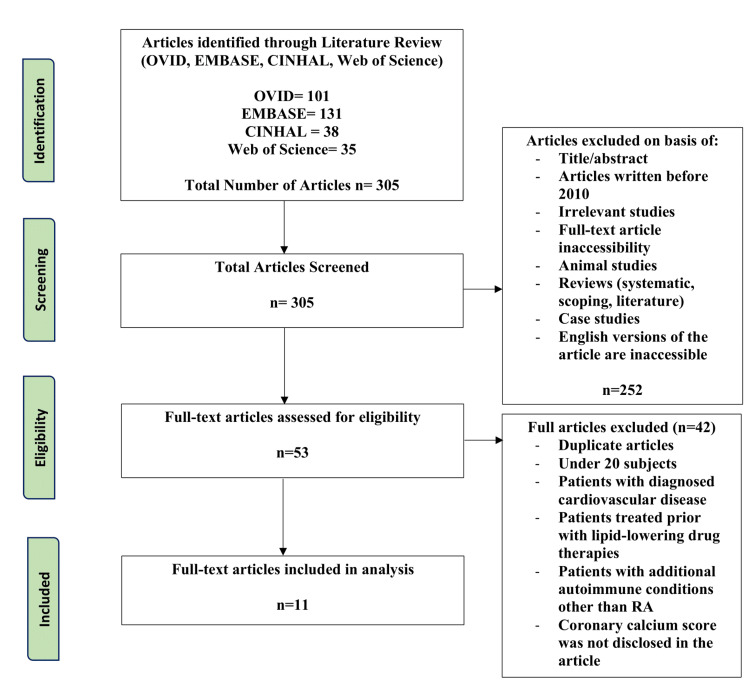
PRISMA diagram PRISMA: Preferred reporting items for systematic reviews and meta-analyses

For this review, we included randomized controlled trials, cross-sectional studies, observational studies, and cohort prospective/retrospective studies. The patient population studies needed to include patients without prior history of CVD diagnoses or treatment with lipid-lowering medications and with a sole autoimmune diagnosis of RA. Studies excluded from this review were designs of literature, systematic or scoping reviews, case studies, animal studies, articles published prior to the year 2010, inaccessible full-text versions, articles without English translation, and duplicate studies. In addition, articles were excluded if they assessed less than 20 subjects in an effort to ensure sufficient power and generalizability of the study, including patients with pre-existing CVD being managed concurrent with their RA, patients with additional autoimmune disorders, and if the CAC of patients was not disclosed within the article. The articles were assessed using the Joanna Briggs Institute critical appraisal checklists [19]. The preferred reporting items for systematic reviews and meta-analyses (PRISMA) were used to develop a flow diagram of the selection criteria for reproducibility (Figure 1) [20].

Results

In total, 305 articles were populated between the four databases (Ovid, Embase, Web of Science, and CINAHL). After the first level of screening, 252 articles were removed based on title, abstract, full-text availability, study type, publication year, and English language availability, with 53 articles eligible for the second round of screening, in which full-texts were completely screened. Articles including patients with subsequent autoimmune conditions, pre-existing CVD, and the use of lipid-lowering medications to ensure no confounding variables were excluded. In addition, duplicate articles and those that did not disclose the CAC score of the patients were removed. Eleven articles were included in the final review, but one article was an 18-month follow-up of the same patients. As a result, 10 unique studies were analyzed in this review.

Table 1 depicts the studies analyzed, including the number of patients, average age, years of disease, CAC score, lipid levels, and medications of patients. In total, 1,141 patients with RA were analyzed, with the majority being females (894, 78.4%) and 247 males (21.6%). The range of ages for patients varied from 53 to 63.4 years of age, with a mean age of 56.9 years. Duration of RA disease was reported in eight out of the 10 unique studies, ranging from six months to 176 months, with a mean of 127.7 months.

**Table 1:** Characteristics of reviewed studies F: Female; M: male; DMARDs: disease-modifying antirheumatic drugs,; HDL: high-density lipoprotein; LDL: low-density lipoprotein; CAC: coronary artery calcium; TNF-α: tumor necrosis factor alpha; IL6: interleukin 6; CTLA4-Ig: cytotoxic T lymphocyte-associated antigen-4-Ig; CD20; JAK: Janus kinase; NSAIDS: nonsteroidal anti-inflammatory drugs; RA: rheumatoid arthritis

Article	Year of publication	# of Patients *	Average age	RA duration in months	CAC score (Agatston units)	Lipid levels	Therapies patients receiving
Montes et al. [21]	2015	124 (90 F, 34M)	59	108 months	CAC score 0: no risk = 40.3% (50 participants); CAC score 1-100: low to moderate risk = 38.7% (48 participants); CAC score 100-400: moderate to high risk = 12.9% (16 participants); CAC score >400: high risk = 8.1% (10 participants)	Total cholesterol: 218 mg/dl; LDL/HDL ratio: 2.1 mg/dl	Biological DMARDs (medications not specified); glucocorticoids
Jesson et al. [22]	2022	50 (41 F, 9 M)	53.7	151.5 months in patients with CAC <100; 163.5 months in patients with CAC >100; average of the two being 157.5 months	CAC score 0: no risk = 50% (25 participants); CAC score 1-100: low to moderate risk = 13% (26 participants); CAC score 100-400: moderate to high risk = 5% (10 participants); CAC score >400: high risk = 7% (14 participants)	Total cholesterol: 210 mg/dl; LDL: 140mg/dl; HDL: 60mg/dl; triglycerides: 130mg/dl	Anti-TNF; anti-IL6; CTLA4-Ig; anti-CD20; anti-JAK; methotrexate; leflunomide; hydroxychloroquine; NSAIDS; corticosteroid
Karpuzas et al. [15]	2013	150 (131 F, 19 M)	53	132 months	Mean total = 84.4 Agatston units; mean of (55) patients with CAC above 0 = 230.1 Agatston units	None reported	Prednisone; methotrexate; TNF-a inhibitors
Tinggaard et al. [23]	2020	395 (289 F, 106 M)	63.4	Not reported	CAC score 0: no risk = 40.3% (159 participants); CAC score 1-100:; low to moderate risk = 29.4% (166 participants); CAC score 100-400: moderate to high risk = 15.4% (61 participants); CAC score >400: high risk = 14.9% (59 participants)	None reported	Conventional DMARDs; biological DMARDs (medications not specified)
Bernardes et al. [24]	2019	60 (60 F, 0 M)	53.6	176 months	CAC score 0: no risk = 50% (30 participants); CAC score 1 – 10 : low risk = 18.3% (11 participants); CAC score 10-100: low-moderate risk = 21.7% (13 participants); CAC score 100-400: moderate to high risk = 8.3% (5 participants); CAC score >400: high risk = 1.7% (1 participants); mean score = 35.192 Agatston units; breakdown within coronary artery calcification; left main coronary artery = 3.445 Agatston score; left anterior descending artery = 16.755 Agatston score; left circumflex artery = 6.198 Agatston score; right coronary artery = 8.797 Agatston score	HDL: 61.07 mg/dl; LDL: 133.23 mg/dl; triglycerides: 117.77 mg/dl; LDL/HDL ratio: 2.367; triglycerides/HDL ratio: 3.768; ApoB/Apo A1 ratio 0.695	Corticosteroids; NSAIDs; methotrexate; Leflunomide; hydroxychloroquine; sulfasalazine; azathiopurine; adalimumab; etanercept (anti-TNF-a); infliximab (anti-TNF-a); rituximab (anti-CD20); anakinra (anti-IL1); tocilizumab (anti-IL6); bisphosphonates; folic acid
Udachkina et al. [25]	2017	74 total, however 12 had ischemic heart disease and were excluded from analysis leaving 62 patients for review (48 F, 14 M)	56	6 months	CAC score 0: no risk = 64.5% (40 participants); CAC score 1-10: low risk = 22% (5 participants); CAC score 10-100: low risk = 50% (11 participants); CAC score 100-400: moderate to high risk = 18% (4 participants); CAC score >400: high risk = 10% (2 participants); mean = 55 Agatston units	Triglycerides: 93. 7 mg/dl; LDL: 61.3 mg/dl; HDL: 23.4 mg/dl	Study was conducted prior to patients receiving DMARDs or corticosteroids
Udachkina et al. [26]	2018	18 month follow up on the 74 patients (48 F, 14 M)	56	24 months	19 out of the 22 patients experienced an increase in CAC score; increase was 21.3 Agatston units-approximate increase of 20% which exceeds the normal annual increase in the general population; mean = 75 Agatston units	Triglycerides: 99.1 mg/dl; LDL: 61.3 mg/dl; HDL: 27.0 mg/dl	Methotrexate; adalimumab (anti-TNF-a); abatacept (blocks T cell stimulation); certolizumab (anti-TNF-a); rituximab (anti-CD20); infliximab (anti-TNF-a)
Liu et al. [27]	2017	49 (44 F, 5 M)	54	156 months	CAC score 0 = 40% (16 participants); CAC score 1-10: no risk = 3% (1 participants); CAC score 10-100: low risk = 22% (9 participants); CAC score 100-400: moderate to high risk = 25% (10 participants); CAC score > 400: high risk = 10% (4 participants)	Cholesterol: 91.9 mg/dl; HDL: 27.0 mg/dl; LDL: 54.1 mg/dl; triglycerides: 23.4 mg/dl	Methotrexate; hydroxychloroquine; sulfasalazine; leflunomide; prednisolone
Winchester et al. [28]	2016	72 (62 F, 10 M). Of the 72, 24 were found to have CAC	54	Not reported	CAC score 0 = 67% (48 participants); CAC score 1-100: low to moderate risk = 15% (11 participants); CAC score 100-400: moderate to high risk = 10% (7 participants); CAC score >400: high risk = 8% (6 participants)	Cholesterol: 193 mg/dl; LDL: 108 mg/dl; HDL: 61 mg/dl	Methotrexate; prednisone; NSAID; TNF inhibitors
Corrales et al. [29]	2014	75 (56 F, 19 M)	60.7	156 months	Patients with RA had significantly greater prevalence of calcification compared to control (65.3% vs. 49.3%); mean CAC score (49 patients) = 197 Agatston units	Cholesterol 220 mg/dl; LDL 130 mg/dl; HDL 70 mg/dl; Triglycerides 110 mg/dl	Methotrexate; leflunomide; sulfasalazine; tocilizumab (anti-IL6); abatacept (blocks T cell stimulation); rituximab (anti-CD20); glucocorticoid; NSAID
Fang et al. [30]	2013	104 (73 F, 31 M)	59.3	130 months	CAC score 0 = 39% (41 participants); CAC score 1-100: low to moderate risk = 40% (42 participants); CAC score 100-400: moderate to high risk = 12% (12 participants); CAC score >400: high risk = 9% (9 participants)	Cholesterol 215 mg/dl; HDL 63.4 mg/dl	Not reported

**Figure 2:**
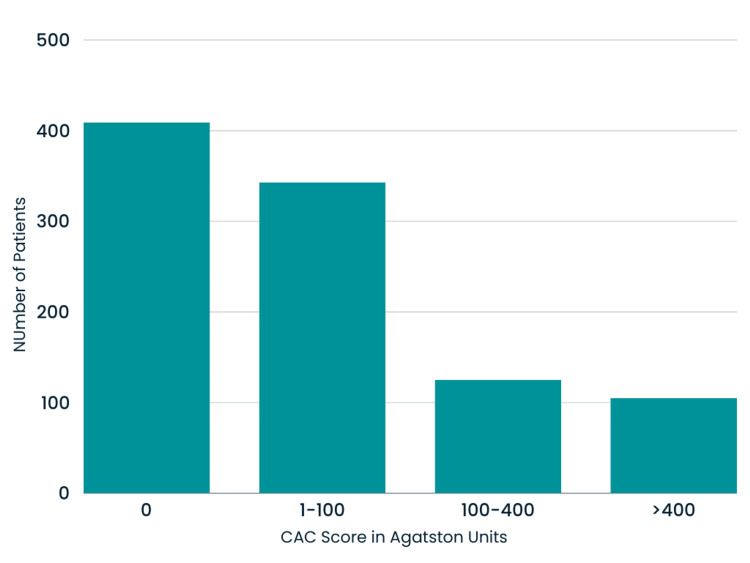
The number of patients within each category of CAC score (Agatston units) CAC: Coronary artery calcium 0 (no risk), 1-100 (mild-moderate risk), 100-400 (moderate-high risk), and above 400 (high risk) [22-30]

**Figure 3:**
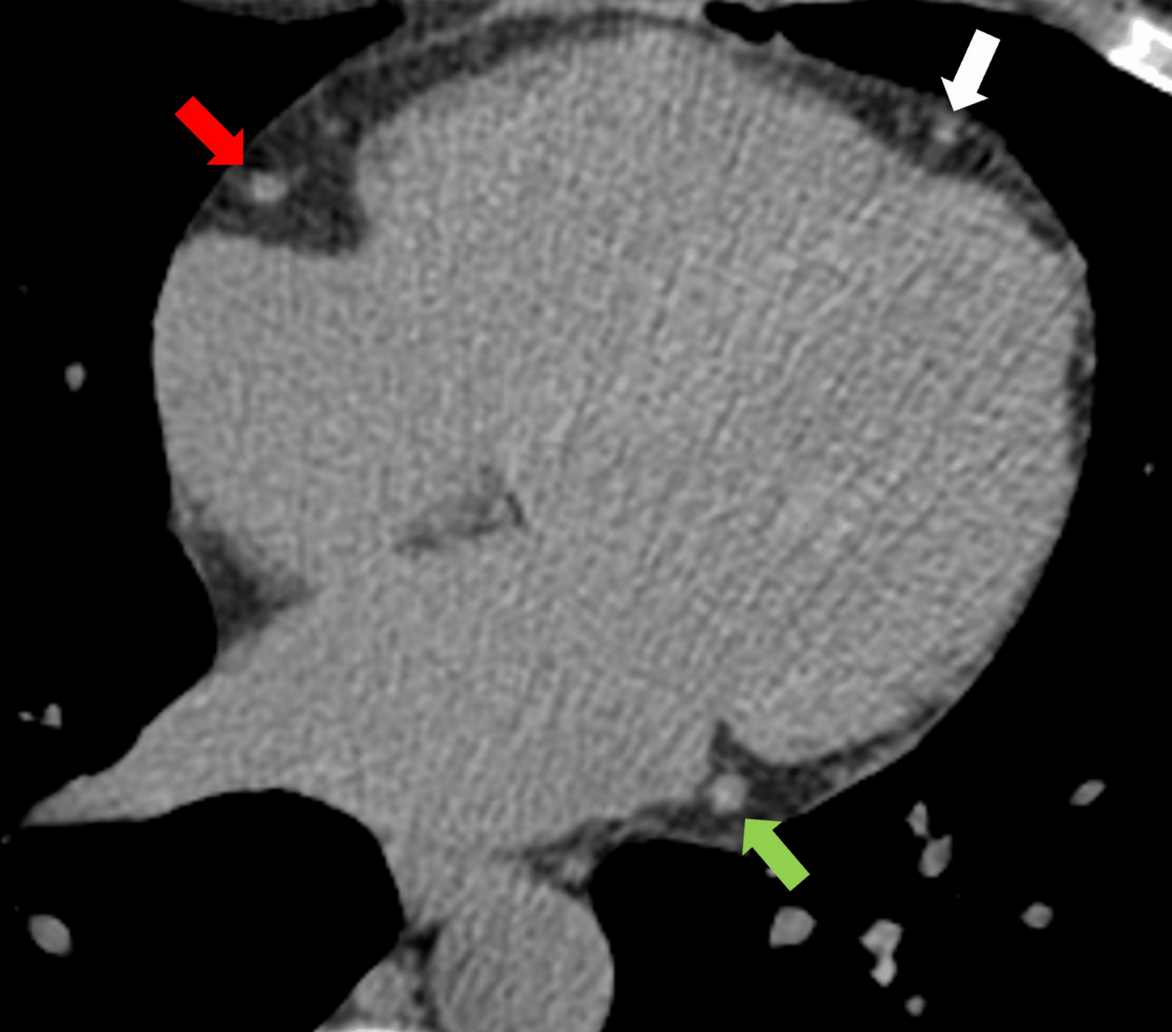
A 51-year-old female with a coronary artery calcium score of zero. Axial non-contrast cardiac-gated CT image of the heart shows no calcified atherosclerotic plaque in the left anterior descending artery (white arrow), the left circumflex artery (green arrow), or the right coronary artery (red arrow) CT: Computed tomography CT images are the property of Dr. Jordan Ditchek

**Figure 4:**
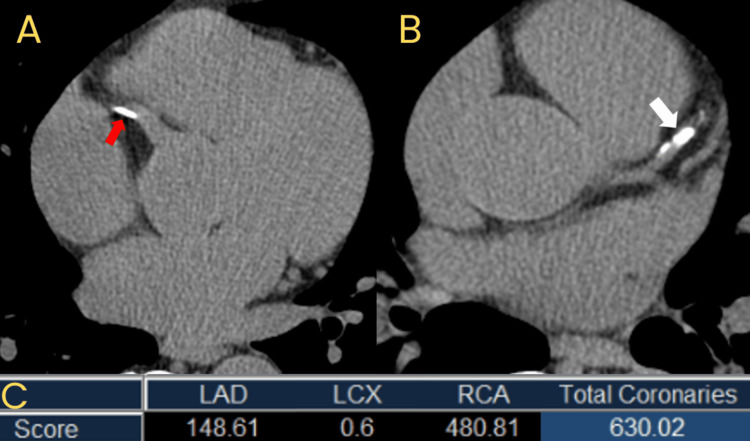
A 69-year-old male with hypercholesterolemia Axial non-contrast cardiac-gated CT images demonstrate calcified atherosclerotic plaque in the LAD (red arrow) (A) and the RCA (white arrow)(B).  Output from the calcium scoring software (C) reports the total calcium score for all calcified plaques in each vessel, and the sum total for all vessels (630 in this patient). This patient’s score falls at the 79th percentile for an age- and sex-matched cohort. CT: Computed tomography CT images are the property of Dr. Jordan Ditchek

Figure 2 represents the findings of eight of the studies that included the breakdown of CAC scores from 0 (no risk), 1-100 (mild-moderate risk), 100-400 (moderate-high risk), and above 400 (high risk). As some studies reported CAC scores of 0-10, 1-10, and 1-100 Agatston units, a decision was made for analysis to include all of these subgroups under the main group of 1-100 Agatston units representing mild-moderate disease states. Karpouza et al. and Paccou et al. were the two studies not included in the analysis due to only providing the mean CAC score found with CT calcium scoring, unlike the other studies, which categorized the groups depending on Agatston score and risk [15,31]. Of the reported studies, 409 patients had a very low-risk CAC score of 0, 343 had a mild-risk CAC score of 1-100, 125 had a moderate-risk CAC score of 100-400, and 105 had a high-risk CAC of over 400. Overall, 59.3% of patients with RA reported an elevated CAC score above 0 Agatston units. To provide visualization of the plaque buildup that can occur and occlude the coronary arteries, Figure 3 represents an individual with no atherosclerotic plaque, and Figure 4 indicates the calcified plaque that can appear during CAC scoring.

Table 2 analyzes the presence of RF and ACCP biomarkers as well as inflammatory markers of C-reactive protein (CRP) and erythrocyte sedimentation rate (ESR). Each study reported that 50% or more of their participants were seropositive for RF, ACPA, or both. RF and ACPA have been shown to have a synergistic role in the inflammatory processes of patients with RA and have been linked to progressing CVD [31]. CRP and ESR levels elevate in response to nonspecific inflammation within the body. The normal level of CRP is below 0.3 mg/L, minor inflammation is 0.3-1 mg/L, moderate inflammation is 1-10 mg/L, and severe inflammation is >10 mg/L [32]. In the studies that reported CRP levels, all RA patients had an elevated CRP level from moderate to severe inflammation, ranging from 1.3 to 38.8 mg/L, with an average of 7.6 mg/L. Similarly, ESR rates elevate with aging. The average age of RA patients in the studies examined is 56.7. For males above 50, the normal value of ESR is ≤20 mm/hr, and for females is ≤30 mm/hr [33]. ESR was less commonly reported within the studies as compared with CRP, and the values ranged from 2 mm/hr to 37 mm/hr. CRP is the preferred method for measuring the levels of inflammation due to it being a direct measurement as compared with ESR, which is an indirect measurement that can be impacted by the size, shape, and number of erythrocytes. It can also be impacted by age, sex, and fibrinogen levels [34]. This may explain the discordance between the number of studies examining CRP compared to ESR as well as providing reasoning between the elevations in CRP indicating inflammation versus the absence or mild elevations in ESR levels.

**Table 2:** Presence of biomarkers/inflammatory markers

Article	# of patients	Rheumatoid factor	Anti-cyclic citrullinated peptide (ACPA)	ESR (<20mm/hr males) (<30mm/hr females)	CRP (<0.3 mg/L)	Additional biomarkers
Montes et al. [21]	124	72 patients positive with a mean titer of 335 U/mL	75 patients positive with a mean titer of 1060 U/mL	12 mm/hr	2 mg/L	Anti-citrullinated fibrinogen 10 patients positive with a mean titer of 2.3; anti- citrullinated fibrinogen-beta 36-52; 29 patients positive with a mean titer of 13.5
Jesson et al. [22]	50	Present in 28 patients with CAC < 100 and 7 with CAC > 100, no exact RF value reported	Present in 25 patients with CAC < 100 and 9 with CAC > 100, no exact ACPA value reported	2 mm/hr with CAC <100; 2.2 mm/hr with CAC >100	2 mg/L with CAC <100; 2 mg/L with CAC >100	
Karpuzas et al. [15]	150	129 patients positive	127 patients positive	27 mm/hr	2.6 mg/L	
Tinggaard et al. [23]	395	Not directly reported. 317 patients were positive for RF and/or ACPA	Not directly reported. 317 patients were positive for RF and/or ACPA	Not reported	Not reported	
Bernardes et al. [24]	60	34 patients positive for RF; Mean titer of 143 total.	48 patients positive for RF; Mean titer of 440.7 total.	Mean 27.98 mm/hr total	Mean 12.5mg/L mm/hr total	
Udachkina et al. [25]	74 total, however 12 had ischemic heart disease and were excluded from analysis leaving 62 patients for review	65 patients positive	74 patients positive	37 mm/hr	38.8 mg/l	
Liu et al. [27]	49	Not reported	Not reported	Not reported	1.3 mg/l	
Winchester et al. [28]	72	Not directly reported. 55 patients were positive for RF and/or ACPA	Not directly reported. 55 patients were positive for RF and/or ACPA	Not reported	2.7 mg/l	IL-6 3.7 pg/ml in patients experience CAC
Corrales et al. [29]	75	58 patients positive	50 patients positive	Not reported	2.7 mg/l	
Fang et al. [30]	104	61 patients positive	60 patients positive	Not reported	Not reported	

Discussion

The association between RA and musculoskeletal changes is well-known and published within the literature. More recently, extrasystemic manifestations including cardiovascular system implications have been a focus of research efforts, with most noting that the impacts are due to increased inflammation tied to RA.

The pathophysiology tied to the development of cardiovascular comorbidities has yet to be fully elucidated, but chronic inflammation tied to autoimmune conditions such as RA has been found to significantly progress atherogenesis. One hypothesis surrounds the accumulation of neutrophil extracellular traps released via neutrophils to eliminate pathogens and reduce inflammation, but this release may also trigger the release of IL-1, inducing further inflammation and leading to endothelial damage and atherosclerosis [35]. In addition, in many autoimmune states, including RA, reactive oxygen specials (ROS) have been shown to be elevated. In the presence of an immune system dysfunction and diminished presence of anti-inflammatory markers, ROS can induce endothelial damage, which leads to plaque buildup [36]. The significant rise of inflammatory markers including TNF-α, IL-1, and IL-6 among RA patients can damage the endothelial lining of the vasculature as well as recruit smooth muscle cells and lipid-laden macrophages into the intima that can result in rapid plaque formation [37,38]. The multifactorial nature of the immune system attacks the coronary vessels on numerous levels, leading to vasculature inflammation, damage, and plaque accumulation. 

Most patients with RA analyzed in this review were positive for RF and/or ACPA, which not only indicates a prominent inflammatory state but can also be tied to independent risk factors of atherogenesis. Research efforts have suggested that citrullinate epitopes within the atherosclerotic plaque are targets for ACPA, leading to autoimmune inflammation in the vessels and further calcified plaque buildup [39]. High ACPA levels are positively correlated with ESR, CRP, IL-2, IL-8, IL-6, IL-17, IL-23, metalloproteases, and ROS and are negatively associated with antioxidants [40]. 

Noninvasive CAC scoring has gained popularity compared to its invasive counterpart of coronary angiography. A normal CAC score is 0, indicating no plaque buildup within the vessels, with anything above 0 indicating an abnormality in the vasculature [16]. Overall, this review revealed that 59.3% of patients with RA reported a positive finding with noninvasive CT coronary angiography. As such, based on these findings, additional studies are warranted to determine whether patients with RA should require proper cardiac screening beginning at the time of diagnosis in an effort to reduce CVD risk, especially among patients with more severe disease. 

Practicing rheumatologists have become more aware of the risk of severe cardiac morbidity tied to RA, especially as the European Alliance of Associations for Rheumatology (EULAR) updated recommendations for cardiac risk assessment in 2022 [10]. The focus should shift away from treating only the musculoskeletal aspects of RA to preventing cardiovascular outcomes. The EULAR committee recommended 10 guidelines for clinicians to manage and prevent the occurrence of CVD in RA patients [10]. The key recommendations include optimally controlling disease states, completing cardiovascular risk assessments every five years, measuring triglyceride and high-density lipoprotein (HDL) levels during stable disease states or remission, multiplying current cardiovascular risk models (i.e., Framingham and/or Reynolds) by 1.5x for RA patients, the development of additional risk assessment models like the Extended Risk Score-RA (ERS-RA) and the Transatlantic Cardiovascular Risk Calculator for RA (ATAAC-RA), screening asymptomatic patients with carotid ultrasound, focusing on lifestyle changes within patients, managing cardiac risk with medications, and caution when prescribing nonsteroidal anti-inflammatory drugs (NSAIDs) and glucocorticoid therapy [13,41-43]). In addition, the use of medications, including statins, proprotein convertase subtilisin/kexin type 9 inhibitors (PCSK9), and even colchicine, have been found to be beneficial in primary and secondary prevention [44-46]. However, surprisingly, even with the EULAR recommendations indicating the required focus, less than half of patients with RA receive lipid screening, and rheumatologists were found to order lipid levels less often than primary care physicians (PCP) for patients with RA, it is hypothesized that rheumatologists may see prevention to be in the realm of PCPs even with the greater understanding of the cardiovascular risks [47,48]). Up to 97% of patients are unaware of the CVD risks, which are associated with their RA diagnosis [49]. This represents the increasing need to educate both physicians and patients about the rising CVD inflammation and outcomes associated with this progressive autoimmune condition.

Another critical area to examine is the differences between the sexes. As with most autoimmune conditions, RA impacts women at a greater rate than men, as over 70% of RA diagnoses are among women, with a peak incidence in the fifth decade of life [50]. This trend is seen within the studies analyzed, as 78.4% of the patients with RA followed this skew and were females. The discrepancies between the sexes have yet to be fully elucidated. Meanwhile, it is crucial to investigate this as the development of coronary inflammation is multifaceted, and female genetics may play a significant role. The hormonal impact of estrogen has been studied and has shown both positive and negative effects among females with RA. Estrogen can limit cardiac risks through a variety of ways, including restricting lipid peroxidation, downregulating inflammatory markers, reducing the embolism of plaque by reducing metalloproteinases, dilating blood vessels, elevating HDL, and lowering low-density lipoprotein (LDL) levels, and overall plays a significant role in balancing the antioxidant and oxidative stress states [51]. During periods of elevated estrogen levels, like pregnancy, females with previously diagnosed RA have been found to have significant symptom regression or go into remission and relapse a few months postpartum as estrogen levels decline [52]. On the other hand, estrogen has also been found to increase inflammation as estrogen receptors are found on numerous immune cells (T cells, B cells, macrophages, and natural killer (NK) cells). Females have been shown to have enhanced production of autoantibodies within B cells and higher antigen-presenting activity [53,54]. The severity of inflammation has been shown to be linked with the progression of coronary calcification, and if females experience elevated inflammation due to estrogen, this may play a significant role in the differences between sexes. On the other hand, testosterone has been shown to increase IL-10, an anti-inflammatory marker. This is further demonstrated in males with androgen insufficiency with higher proinflammatory markers and greater T and B cell activity [55,56]. In addition, the average age of participants in the studies analyzed was 56.9. As the majority of RA patients are females above age 50, it is important to recognize the duality of RA inflammation and the loss of estrogen in postmenopausal women. With the loss of estrogen’s protective factors, postmenopausal women with RA are at an even higher risk for the development of coronary artery disease. As such, additional research is warranted to determine if CT calcium screening should be considered in this high-risk group to mitigate the risk of negative CVD outcomes. 

While some medications provided to RA patients aimed at reducing inflammation and stabilizing disease state, these same medications are also associated with the risk of CVD. The patients analyzed within the studies were placed on various glucocorticoids, biologic disease modifying antirheumatic drugs (DMARDS), and conventional DMARDs. DMARDs are powerful anti-inflammatory agents that lower lipid levels and reduce CVD risk. Meanwhile, glucocorticoids, a medication known to reduce inflammation adequately, have been shown to increase CVD risk, including hypertension and dyslipidemia, as they increase very-low-density lipoprotein (VLDL), cholesterol, and LDL, as well as reduce HDL [57]. Patients receiving higher levels of glucocorticoids had a 47% increase in CVD events as compared with patients with RA who did not receive them [58]. In addition, patients with seropositive RA have been shown to exhibit triple the risk for CVD with the use of glucocorticoids [57]. Glucocorticoid dosages and length of treatment must be monitored to balance the anti-inflammatory nature and limit CVD risk, as the EULAR recommends.

A limitation to analyzing CAC scoring to determine calcification within the coronary vessels of patients with RA is that soft plaque or low-attenuated plaque (LAP) containing no calcium is overlooked. LAP can pose a greater risk than the more commonly analyzed calcified plaque. Those with LAP have significantly greater plaque progression, 3-5 times the risk of heart disease and myocardial infarction, double the risk of developing artery narrowing, and 10-fold increase in CVD when LAD was combined with pre-existing artery stenosis [59,60]. As a result, it is recommended to monitor patients with RA for CVD, even those with low or no CAC score, due to the hidden damage of LAP.

A key limitation to the study is inconsistent reporting of CAC score. Most studies reported the classification of CAC score in a range, very few reported the mean CAC score, and none reported individual scores. As a result, it was unclear the exact CAC score of each patient and their characteristics to better understand the potential trends behind the severity of plaque accumulation. In addition, the anti-inflammatory function of DMARDs may mask the true CVD risk for patients with RA. Furthermore, the CVD risk rises with the length of the disease. The wide range of six to 176 months may impact the results found, as those with early disease states have not experienced the same number and duration of flare-up and their risk for CVD may be thought to be lower.

## Conclusions

Examining the inflammatory effects of RA is crucial to understanding its systemic effects well beyond the joint synovium. Almost 60% of patients with RA showed the presence of coronary calcification, which is well above the expected range for age-matched healthy controls without RA. As such, patients with RA likely carry a higher risk for adverse cardiovascular outcomes as compared to their healthy counterparts. Additional research is warranted based on these findings to determine whether the addition of CT coronary angiography and analysis of laboratory markers for CVD, including lipid markers in standard protocols for RA comorbid assessment, would help to reduce adverse cardiovascular complications. As CVD is the leading cause of morbidity and mortality among RA patients, it is critical for rheumatologists to become proactive in preventing these negative CVD outcomes within this at risk population.
